# Importance of EPA and DHA Blood Levels in Brain Structure and Function

**DOI:** 10.3390/nu13041074

**Published:** 2021-03-25

**Authors:** Clemens von Schacky

**Affiliations:** Omegametrix, Martinsried, Am Klopferspitz 19, 82152 Martinsried, Germany; c.vonschacky@omegametrix.eu; Tel.: +49-89-5506-3007; Fax: +49-89-5506-3008

**Keywords:** eicosapentaenoic acid, docosahexaenoic acid, omega-3 index, biomarker

## Abstract

Brain structure and function depend on a constant and sufficient supply with eicosapentaenoic acid (EPA) and docosahexaenoic acid (DHA) by blood. Blood levels of EPA and DHA reflect dietary intake and other variables and are preferably assessed as percentage in erythrocytes with a well-documented and standardized analytical method (HS-Omega-3 Index^®^). Every human being has an Omega-3 Index between 2 and 20%, with an optimum of 8–11%. Compared to an optimal Omega-3 Index, a lower Omega-3 Index was associated with increased risk for total mortality and ischemic stroke, reduced brain volume, impaired cognition, accelerated progression to dementia, psychiatric diseases, compromises of complex brain functions, and other brain issues in epidemiologic studies. Most intervention trials, and their meta-analyses considered EPA and DHA as drugs with good bioavailability, a design tending to produce meaningful results in populations characterized by low baseline blood levels (e.g., in major depression), but otherwise responsible for many neutral results and substantial confusion. When trial results were evaluated using blood levels of EPA and DHA measured, effects were larger than comparing EPA and DHA to placebo groups, and paralleled epidemiologic findings. This indicates future trial design, and suggests a targeted use EPA and DHA, based on the Omega-3 Index.

## 1. Introduction

Among other parameters, like blood glucose, brain function depends on brain structure, brain perfusion, and, in some cases, on the absence of inflammatory processes [1,2,3]. Additionally, the fatty acid composition of a cell’s membrane impacts on physical stability, signal transduction, ion channel behavior, and many other cell properties and functions [1,2]. Here, current evidence on the relevance of EPA and DHA to brain is reviewed. The perspective of blood levels of EPA and DHA is used, preferably when measured in erythrocytes (Omega-3 Index) [4,5]. The Omega-3 Index is a long-term parameter, correlates with EPA and DHA in other cells in the body thus far studied, and therefore reflects an individual’s EPA and DHA status [4,5].

## 2. Relevance of EPA and DHA to the Brain

The Relevance of EPA and DHA to the brain can be broadly categorized as follows:

*Building and Maintaining Brain*: Conversion of plant-derived alpha-linolenic acid to DHA is minimal in humans, as is formation of DHA from EPA [5,6]. Therefore, preformed DHA is preferred for the build-up of brain during pregnancy and well into the third decade of life, although pregnant women can mobilize stored DHA, and women can increase DHA synthesis estrogen-dependently by some 10% [7,8,9,10,11]. Like all other tissues, brain has a constant cell turnover or apoptosis, albeit a slow one, and therefore, DHA is also needed for brain structure maintenance [7]. Thus, optimal brain structure, and thus function, depends on a life-long supply with DHA to the brain [7,8].

*Brain Perfusion*: Clearly, brain function does not only depend on brain structure, but also on brain perfusion. The brain uses some 20% of the blood pumped by the heart, with white matter receiving some 25, and gray matter receiving some 90 mL/100 g/min [12]. Brain perfusion is distributed by arteries and arterioles, which are modulated by vasoactive molecules, some of them derivatives of EPA, and some of them derivatives of DHA. Therefore, it is no surprise that a higher Omega-3 Index was found to be related to higher regional cerebral blood flow on brain Single Photon Emission Computed Tomography [13]. In addition, blood vessels can be obstructed, e.g., by an embolus or by rupture of an arterial plaque, resulting in an ischemic stroke. Both risks for embolism and plaque rupture are less at higher blood levels of EPA and DHA as compared to lower blood levels [14,15,16,17]. Finally, a hemorrhagic stroke results from brain vessel rupture, e.g., due to arterial malformations or elevated blood pressure. EPA and DHA lower blood pressure [18]. Both kinds of stroke can result in catastrophic losses of brain structure.

*Inflammatory Processes in Brain*: Some issues in brain function result from regional inflammatory processes, like major depression [19]. Both EPA and DHA reduce severity of inflammation, with mechanisms and/or metabolites mitigating development of inflammation, as well as facilitating resolution of inflammation [20].

Clearly, the three categories overlap to degrees that are hard to define when it comes to brain function. However, just as clearly, for optimal brain function, an optimal supply of EPA and DHA is needed on a constant basis. The only source of EPA and DHA for the brain is the blood stream. Uptake of DHA from the bloodstream into the brain, however, can be an active process, with the genetic trait ApoE4 playing a role, among others [21,22]. Taken together, it makes more sense to assess these two fatty acids in the blood stream, than in the diet, to study the relationships EPA and DHA with brain structure and function.

## 3. Dietary Intake vs. Blood Levels of EPA and DHA

Nevertheless, many epidemiologic studies, many intervention trials, and many previous reviews (systematic or not), related dietary intake of EPA and DHA to brain issues.

Typically, in epidemiologic studies, food intake was assessed using food frequency questionnaires. This approach has been criticized for producing some 50% of implausible data [23]. After assessing dietary intake of specific foods, e.g., salmon (an established source of EPA and DHA), salmon concentration of EPA and DHA is looked up in time-honored tables. This approach disregards the fact that, in recent years, the concentration of EPA and DHA in salmon decreased by 50% [24]. Therefore, epidemiologic studies relating dietary intake to brain issues are prone to large systematic and non-systematic errors. This explains why epidemiologic studies relating blood levels of EPA and DHA to clinical events or parameters usually found stronger correlations than those assessing dietary intake, e.g., [25,26,27]. An example is blood pressure, which related inversely to blood levels of EPA and DHA, but not to their dietary intake [28,29].

Typically, for intervention trials, participants were recruited based on clinical criteria, but irrespective of baseline blood levels of EPA and DHA [30,31]. Then, participants were randomized to receive either a fixed dose of EPA and DHA or a placebo, analogous to a drug trial, where the absence of a drug is compared to its presence [30,31]. However, all humans have blood levels of EPA and DHA that have a statistically normal distribution in all populations studied thus far [5]. Of course, no effect of EPA and DHA can be detected in individuals with optimal blood levels; rather, a deficit at baseline is a prerequisite for an effect to be demonstrated. This explains why trials in health issues characterized by a low Omega-3 Index, like major depression or congestive heart failure with reduced ejection fraction were more likely to demonstrate effects than trials in multifactorial health issues, like cardiovascular disease or impaired brain function [30,31]. Therefore, a drug trial design cannot be used uncritically for EPA and DHA, and baseline blood levels of EPA and DHA must be included in the in- or exclusion criteria, as must be the blood levels reached during the trial due to the complex bioavailability of EPA and DHA.

Many large intervention trials ignored issues of bioavailability of EPA and DHA [30,31]. Many trials were executed by clinical research organizations, routinely instructing trial participants to take trial medication in the morning, e.g., with breakfast. Frequently, breakfast, if taken at all, is a low-fat meal [30,31]. Since absorption of EPA and DHA requires fat digestion, and fat digestion needs to be initiated with sufficient fat in a meal, bioavailability of EPA and DHA was thus minimized [30,31,32]. Moreover, inter-individual variability in bioavailability is large (factor 13), which, together with the statistically normal distribution of the baseline blood levels unaccounted for, leads to blood levels of EPA and DHA overlapping during a trial between Verum (i.e., EPA and DHA) and Placebo groups [5,30,31,32,33]. Since clinical events correlate more closely with blood levels than with dietary intake of EPA and DHA, a sufficiently large overlap of blood levels of EPA and DHA between EPA and DHA and Placebo groups will make it impossible to discern a difference in number of clinical events between EPA and DHA and Placebo groups [5,30,31,32]. As many systematic reviews and meta-analyses disregarded the methodological issues just discussed, and included trials based on other criteria, misleading results were and are being provided.

More recently, some trials measured blood levels of EPA and DHA. These trials were then evaluated in two ways: EPA and DHA vs. Placebo and blood levels of EPA and DHA vs. clinical events, e.g., [34,35,36]. Comparing the results demonstrated that effects or larger effects were demonstrated by evaluating blood levels EPA and DHA vs. clinical events than comparing EPA and DHA to Placebo groups.

However, measuring blood levels has its own problems. Blood levels of EPA and DHA measured in plasma or plasma phospholipids rather reflect short-term dietary intake, while other blood levels, e.g., in erythrocytes, rather reflect other cells in the body, and dietary intake is only one of many determinants of blood levels measured. Pre-analytical issues exist, as storage at −20 °C leads to loss of measurable EPA and DHA in erythrocytes within days, while EPA and DHA are more stable, when erythrocytes are stored at room temperature or above freezing point in a fridge [5,37]. When stored at −80 °C, EPA and DHA in erythrocytes are stable for many years [5,37]. Additionally, only one standardized analytical procedure has been validated by extensive scientific publications [5]. Other analytical procedures of erythrocytes often provide vastly different results and have no proper scientific basis [5].

Taken together, in the past, many trials either detected no effects or seriously underestimated the magnitude of the effects of EPA and DHA by comparing clinical events occurring in the EPA and DHA group to clinical events occurring in Placebo, instead of using a level-based approach to trial design and evaluation of the data generated. This is supported by the observation that the magnitude of effects observed in trials evaluating blood levels of EPA and DHA usually resembles the magnitude found in epidemiologic studies, e.g., [26,27,35,36].

## 4. Early Life

### Pregnancy and Lactation

In pregnancy, the placenta preferentially transports AA and DHA towards the fetus, with the aim of achieving slightly more than 8% DHA in fetal erythrocytes [38,39,40]. Thus, it is fair to speculate that slightly more than 8% DHA in erythrocytes is a target preset by nature for the fetus. Clearly, AA and DHA are preferentially transported across the placenta at the expense of the pregnant women [38,39,40]. In an epidemiologic study in Germany, it was found that most pregnant women had an Omega-3 Index below the target range, and that, after giving birth, the Omega-3 Index of the lactating women was even lower, as had to be expected in view of the placental transport just mentioned [41]. Only 11.5% of the pregnant women supplemented Omega-3 fatty acids, which led to a higher mean Omega-3 Index than in non-supplementing women [41]. However, their mean Omega-3 Index still was below the target range, and, more importantly, individual Omega-3 Index values varied almost as widely as in non-supplementing women [41]. The consequences of an Omega-3 Index below the target range of 8–11% in pregnancy are reviewed in detail elsewhere [8]. Among them are non-brain related complications, like increased rates of premature birth, perinatal infant mortality, or intensive care for the mother [8]. Brain-related complications are perinatal depression of the mother, and issues of brain structure and cognitive development in the child [8].

There is little doubt that a low Omega-3 Index during pregnancy is concentration-dependently associated with risk for perinatal depression [42,43,44]. Increasing dietary intake of EPA and DHA, preferably with a product containing more EPA than DHA, is safe in pregnant women and improves perinatal depression, although not all meta-analyses agree on the efficacy, probably illustrating the methodologic issues discussed above [45,46,47,48].

Babies born to mothers supplementing with DHA and AA during pregnancy were found to have larger brains, as measured by magnetic resonance than babies born to mothers ingesting placebo in a randomized controlled trial [49]. Similar findings were reported from an epidemiologic study [50]. Aspects of brain function of the offspring were seen to be improved in some, but not all trials [51,52,53,54,55,56,57,58].

Some, but not all current pertinent guidelines recommend EPA and DHA in pregnancy. The Australian Pregnancy Care Guidelines, endorsed by National Health & Medical Research Council state as evidence-based, “advise pregnant women that supplementation with omega-3 long-chain polyunsaturated fatty acids (800 mg DHA and 100 mg EPA per day) may reduce their risk of preterm birth, if they are low in omega-3.” [59]. Thus, this Australian guideline recommends assessing a pregnant woman’s status in EPA and DHA to target supplementation with EPA and DHA [59]. However, in other countries, like in Germany, irrespective of baseline status, supplementation with 200 mg/day DHA is recommended by some but not all scientific societies [8]. As discussed in more detail above and elsewhere, a targeted supplementation with EPA and DHA in pregnancy appears more effective and safer than an untargeted one [8].

Mothers with a low Omega-3 Index have a higher likelihood to have a pre-term birth, and brain is built up in the last trimester [8]. The brain stem is formed very early in pregnancy [10] and accrues DHA in the last trimester [8]. Babies born pre-term have a lower Omega-3 Index than babies born at term [60]. Thus, babies born pre-term need additional DHA, and probably also AA, for proper brain development. Indeed, a meta-analysis of pertinent intervention trials demonstrated the positive effects of DHA and AA on aspects of brain function in 12–24 months old infants born pre-term [55]. This puts neutral findings of a more recent trial and an older Cochrane Meta-Analysis into perspective [61,62].

In infants born at term, at 12 months of age, red cell DHA content was found to correlate with problem solving behavior [63]. The most important trial in term infants probably is the DIAMOND trial, which tested infant formulas containing no, 0.32%, 0.64%, and 0.96% DHA for 12 months in a total of 343 participants; formulas containing DHA also contained 0.64% AA [64]. The trial was completed by 244 participants. Both at 4 and 12 months, red blood cell DHA increased dose-dependently [64]. Both at 4 and 12 months, however, red blood cell AA also increased with increasing doses of DHA up to 0.64% but was markedly lower with 0.96% DHA [64]. Brain function parameters measured at six years of age, like attention span or verbal or full-scale intelligence quotient followed the pattern of AA in red blood cells [65]. At nine years of age, with magnetic resonance imaging, brain structure-function relationships were also studied, with results for connectivity or task activation again following the pattern of AA in red blood cells [66]. Thus, the results of the DIAMOND trial are a strong argument for controlling red blood cell levels of DHA and AA, in order to avoid reducing red blood cell levels of AA, and thus availability of AA to the brain. In another 12 months trial in healthy 12 month olds supplementing 100 mg DHA and 100 mg AA, language skills were improved in correlation with both AA and DHA red blood cell phosphatidylethanolamin [67]. A Cochrane-Review of 15 RCT’s with 1889 participants evaluating effects of DHA and AA supplemented versus non-supplemented formula milk on visual function and neurodevelopment reported overall no effect [68]. Again, this was probably due to methodologic issues in design of the original trials discussed above, specifically due to recruiting trial participants without determining baseline and on trial levels of EPA and DHA [68].

## 5. Children and Adolescents

The relationship between the Omega-3 Index and improvements in parameters of cognition in individuals between four and 25 years of age, as investigated in randomized controlled trials has recently been systematically and competently reviewed [3]. Thirty-three trials were included, of which 21 were conducted in typically developing participants, and 12 in participants with a disorder, like autism or attention-deficit-hyperactivity disorder [3]. The authors concluded that, “daily supplementation with ≥450 mg DHA + EPA per day and an increase in the Omega-3 Index >6% makes it more likely to show efficacy on cognition in children and adolescents.” [3]. This conclusion is supported by more recent findings: In children both older and younger than 4 years with undernutrition, a food supplement containing 171 mg EPA and 255 mg DHA per day, and many other components, improved brain blood flow, and parameters of complex brain function, like executive function in a 23-week randomized intervention trial [69]. The latter findings also point towards the positive effects of supplementing with EPA and DHA in children diagnosed to have a deficit by determining a low Omega-3 Index.

### 5.1. Autism and Related Disorders

In addition to the topics discussed in the review just mentioned [3], in autism and autism spectrum disorders, low blood levels of AA, EPA and DHA have been found, according to a meta-analysis of pertinent epidemiologic studies [70]. When intervention trials were meta-analyzed, it was demonstrated that aspects of autism, i.e., social interaction, communication, and repetitive and restrictive interests and behavior can be improved by supplementing EPA and DHA [70]. The latter findings were confirmed in a more recent meta-analysis and a controlled intervention trial [71,72].

### 5.2. Attention-Deficit Hyperactivity Disorder (ADHD)

The review mentioned had a focus on attention-deficit hyperactivity disorder (ADHD), and reported that half of the 12 intervention trials reported positive results but did not clearly recommend EPA and DHA for improvement of aspects of ADHD [3]. Another look at the literature results in a slightly more positive picture. In a meta-analysis of 9 epidemiologic studies, ADHD was associated with low blood levels of EPA and DHA [73]. In a later epidemiologic study, DHA correlated positively with attention, and negatively with severity of ADHD [74]. A meta-analysis of 17 intervention trials found symptoms of ADHD decreased after treatment with EPA and DHA [73]. Similar results were reported in a later meta-analysis [75]. More recently, a 12-week trial with EPA found EPA, but not DHA, increased in red cells, which improved cognitive symptoms more in participants with low baseline EPA than in participants high baseline EPA in red cells [76]. Even more recently, an open label study found the overall ADHD rating scale and several aspects improved after 12 weeks of 500 mg EPA and DHA [77]. Moreover, two trials found treatment with EPA and DHA to be equivalent to treatment with the standard drug, methylphenidate, but with a slower onset of action [78,79]. Importantly, EPA and DHA were found to be effective, in case methylphenidate was not [80]. Although a Cochrane meta-analysis of 2016 did not recommend EPA and DHA in the treatment of ADHD, other systematic reviews and meta-analyses reached more positive conclusions [81,82,83]. A very recent one concluded that, “a combination of eicosapentaenoic acid (EPA) + docosahexaenoic acid (DHA) ≥ 750 mg/d, and a higher dose of EPA (1200 mg/d) for those with inflammation or allergic diseases for duration of 16–24 weeks” would be beneficial in ADHD and that, “the current review also suggested that n-3 index and inflammation may be potential treatment response markers for youth, especially in ADHD and MDD, receiving n-3 PUFA.” [84].

### 5.3. Major Depression in Adolescents

A low Omega-3 Index was found in adolescents with major depression [85]. A similar, more recent study in adolescents found no association of red blood cell DHA levels with depression severity or verbal memory performance, but with red blood cell EPA [86]. An intervention trial in largely non-depressed adolescents with krill oil saw no improvement in depressive symptoms in adolescents, due to issues in compliance: an increase of the Omega-3 Index was seen only in few participants [87]. Another intervention trial in 60 depressed adolescents saw significant improvement, however [88]. Similar, but less pronounced, results were seen in another trial with 56 participants [89]. These positive trials question the overall neutral result of an earlier meta-analysis of four trials in a total of 153 participants [90]. A recent review therefore recommended, “a combination of EPA + DHA of 1000–2000 mg/d, with EPA:DHA ratio of 2 to 1, for 12–16 weeks” for treatment of major depression in adolescents and suggested following treatment response with the Omega-3 Index [84].

Taken together, the current state of the evidence suggests searching for a possible deficit in EPA and DHA in adolescents with autism and related disorders, ADHD, and major depression. In the likely case of diagnosing one by finding a low Omega-3 Index, EPA and DHA should be supplemented in a targeted manner in order to compensate this deficit.

## 6. Brain Damage

*Stroke*: A higher Omega-3 Index was found to be associated with lower risk for ischemic stroke [27,91]. In the Framingham Study, an Omega-3 Index < 4.2% was used as a reference (risk 1.0), while an Omega-3 Index > 6.8% was associated with a risk for ischemic stroke of 0.47 (95% confidence interval 0.21–1.06, *p* for trend across the quartiles reported 0.006) [27]. In the REDUCE-It trial, comparing the EPA and DHA group to placebo, the hazard ratio for ischemic fatal or non-fatal stroke was 0.72 (95% CI 0.55–0.93; *p* = 0.01 [34]. Rates of hemorrhagic stroke were overall low, and not significantly different between EPA and DHA and Placebo groups [34]. However, when the incidence of all strokes was related to serum levels of EPA, all strokes were reduced by some 50% in the same trial [35]. Of note, the reduction in stroke was maximal at mid-high serum levels, allowing the speculation that these blood levels correspond to an Omega-3 Index in the target range of 8–11%, although this remains to be demonstrated directly [35]. Thus, the example of REDUCE-It serves to illustrate the value of measuring omega-3 fatty acid blood levels in trials and analyzing trial results from the perspective of blood levels reached. Of note, the results of the intervention trial REDUCE-It were remarkably similar to the results obtained in the Framingham study by measuring the Omega-3 Index [27]. Therefore, an Omega-3 Index in the target range provides potent prevention from stroke.

*Injury*: Some athletes, like boxers, American football or soccer players are subject to multiple traumatic brain injuries during their careers, resulting in white matter lesions and diseases like major depression or issues in cognition [92]. Minor head traumas can also occur in everyday life, however. In a randomized controlled trial, increasing blood levels of DHA reduced blood levels of neurofilament light in American football players [93,94]. Neurofilament light is a biomarker that reflects axonal damage, and therefore a reduced release of this biomarker indicated that, with higher blood levels of DHA, consequences of brain injury were mitigated [93,94]. These results complement similar findings in animal studies, which is why Omega-3 fatty acids are considered a promising approach towards preventing damages and/or improving outcomes following traumatic brain injury [95].

*Fine particulate matter*: Exposure to fine particle matter is thought to be a risk factor for cognitive decline [96]. A higher Omega-3 Index attenuated the inverse associations between fine particle matter exposure and white matter volumes in total brain and a number of brain areas [96]. This is probably mediated by the known anti-inflammatory effects of EPA and DHA and by their positive effects on coagulation and endothelial function [97]. Thus, the benefits of a higher Omega-3 Index may include the protection against detrimental effects of air pollution on brain [96,97].

Other mechanisms of brain damage exist. In Multiple Sclerosis, an inflammatory and demyelinating disease, blood levels of EPA and DHA might be lower than in healthy controls [98]. Low dietary intake of omega-3’s may be an important risk factor for Multiple Sclerosis [99]. Meta-analyses of intervention trials did not find consistent results, with a neutral Cochrane meta-analysis, e.g., [100,101].

Taken together, the data indicate that a high Omega-3 Index prevents stroke and makes brain more resistant to the consequences of common damages by trauma or fine particles.

## 7. Brain Health in Adulthood–Cognition and Dementia

Throughout life, from age four years to the second half of the eighties, at all ages investigated, complex cognitive functions, like problem solving behavior, executive function, or aspects of memory correlated with the Omega-3 Index [102,103,104,105,106,107]. Similar results were found, when blood levels of EPA and DHA were assessed differently, and for DHA in a recent meta-analysis [108,109,110,111]. A low Omega-3 Index predisposes to a more rapid cognitive decline and an earlier onset of dementia compared to a higher Omega-3 Index [105,112,113].

A relatively simple way of measuring complex brain function is assessing reaction time, since this time sums up the time a sequence of complex brain functions takes. In the review already mentioned, trial results were not uniform, but in most trials, an improvement in reaction time was demonstrated [3]. More recently, positive results were published from a trial in healthy 8–9-year-old children [114].

Many intervention trials have been conducted to study the effects of supplementation with EPA and/or DHA on other parameters of brain function, e.g., cognition. A host of pertinent systematic reviews and meta-analyses have been published, most of them with neutral results, e.g., [115,116]. Some, however, found a mild benefit, e.g., [117]. A systematic review of intervention trials found Omega-3 fatty acids to be beneficial at early onset of Alzheimer’s disease, but not in later stages [118]. When intervention trials on parameters of cognition published up to 2015 were broken down according to dose of DHA used, it became apparent that trials using more than 600 mg DHA per day had positive results in terms of aspects of memory, executive function, or aspects of learning, while those using lower doses largely had neutral results [12]. However, more recently, both neutral and positive results were published with doses >600 mg DHA per day [119,120,121,122,123,124,125]. One trial found the improvement in executive function and verbal fluency to correlate with the increase of EPA in red cells, as well as improvements in pertinent brain structures [126]. Another trial found improvements in aspects of memory to correlate with an increase in the Omega-3 Index [127]. Importantly, in one of the trials mentioned, “age-dependent” loss of gray matter volume was reduced by two thirds in the EPA and DHA (Verum) group, as compared to the Placebo group [126]. This demonstrated that two thirds of the “age-dependent” loss of gray matter in the placebo group was a symptom of a deficit in EPA and DHA [126]. Carriers of the genetic trait ApoE4 had less increase of DHA in plasma after dietary intake and only one third uptake of DHA into the brain, compared with non-carriers [128,129]. Additionally, important interactions between status in vitamin B and homocysteine and the effects of Omega-3 fatty acids on cognition have been observed [130,131,132]. The current state of the evidence indicates that many trials not only had some or all of the issues in methodology discussed above, but also ignored potential confounders of the effects of Omega-3 fatty acids, like ApoE4, vitamin B status, homocysteine, and maybe others. Therefore, in the future, trials on cognition need improved designs along these issues to better translate the epidemiologic studies and the lessons from the trials with positive results into clinical evidence. Moreover, the individual omega-3 status should be assessed to determine whether or not an individual is deficient in omega-3 [7,108,111]. Thus, cognitive decline can be slowed, and Alzheimer’s disease can be prevented or improved in its early, but not in later stages of Alzheimer’s disease [133].

A meta-analysis of intervention trials found duration of migraine attacks to be reduced by Omega-3’s, but no effects on frequency and severity [134].

## 8. Psychopathology

Many psychiatric disorders, like major depression or schizophrenia, are neurodevelopmental in nature, i.e., are clinical signs of improper brain development, e.g., [135,136]. As mentioned above, brain development continues into the third decade of life, and depends on availability of EPA and DHA, just like brain maintenance [7]. Therefore, a deficit in EPA and DHA can manifest as a psychiatric disorder. In keeping, when the Omega-3 Index was determined in 131 patients entering a psychiatric residential inpatient clinic, a mean Omega-3 Index of 3.2% was found, indicating that a low Omega-3 Index might predispose to psychiatric diseases [137].

In patients with major depression, a low Omega-3 Index has consistently been found [138,139,140,141,142,143,144,145,146,147]. Moreover, the probability of suicide depended on the concentration of DHA in erythrocytes [148]. Improvements in major depression have largely been associated with increases in the Omega-3 Index [87,149,150,151]. Meta-analyses of intervention trials generally found positive effects [152,153], with the exception of a Cochrane meta-analysis [154]. It is therefore no surprise that EPA and DHA are recommended in some, but not all guidelines [155,156], and that the Omega-3 Index is also mentioned [156].

In patients with bipolar depression, a low Omega-3 Index has been found in most, but not all studies [157,158,159]. Initial meta-analyses of intervention trials were positive, but more recently, the evidence was not seen that positive [160,161,162,163]. A guideline recommending Omega-3’s for bipolar depression could not be found.

In schizophrenia, low blood levels of EPA and DHA have been found [164,165]. Moreover, people at high risk of psychosis have low EPA and DHA levels and low EPA and DHA levels precede the development of psychosis [166]. Evidence from intervention trials with EPA and DHA suggested that transition to psychosis could be prevented, especially in patients at high risk [164]. Results from trials conducted in patients with schizophrenia were not homogeneous [164].

In borderline disorder, some aspects of this personality disorder can be improved according to a recent systematic review and a Cochrane meta-analysis [164,167]. A guideline recommending Omega-3’s for borderline disorder could not be found.

Two of three intervention trials found that EPA and DHA reduce symptoms of post-traumatic stress disorder, and that EPA and DHA can be used for the secondary prevention of this issue of brain function [162].

In occupational burnout, two randomized placebo-controlled intervention trials were conducted. One found an improvement in the Hospital Anxiety and Depression Scale after 52 weeks of 1200 mg EPA plus 600 mg DHA/day in nurses in Japan [168], while the other found improvements in emotional exhaustion, depersonalization decreased, and sense of personal accomplishment increased after 8 weeks of 180 mg EPA plus 120 mg DHA/day for 8 weeks [169].

In other psychiatric diseases, results obtained so far were inconsistent, and research is ongoing [162,164].

Anger, hostility, and components of the Brown’s Attention Deficit Disorder Scales were found to correlate inversely with the Omega-3 Index in a study in an Australian prison [170]. Aggression and psychopathology were found to correlate inversely with the Omega-3 Index in an epidemiologic study from a forensic psychiatric institution [171]. Meta-analyses of pertinent intervention found EPA and DHA to reduce aggression and anti-social behavior in children and adults [164,172,173].

Anxiety correlated inversely with the Omega-3 Index, while mental toughness correlated directly [174].

## 9. Safety and Tolerability

EPA and DHA are considered safe up to 5 g/day according to the European Food Safety Authority, and up to 3 g/day according to the US-American Food and Drug Administration [5,8]. Both in the JELIS and in the REDUCE-It trials, hemorrhagic events (cerebral, fundal, epistaxis, subcutaneous) occurred 0.1% more often in the intervention groups than in the placebo/control groups [34,175]. Both trials were conducted with relatively high doses of EPA, which makes it safe to assume that some participants reached extremely high blood levels. Therefore, we advise against an Omega-3 Index > 16% [5]. In all large cardiovascular trials, EPA and DHA were well tolerated, with fishy eructation being the most frequent complaint. This and other gastrointestinal disturbances can be minimized by ingesting EPA and DHA with the main meal, which, as mentioned, has the advantage of maximizing bioavailability.

## 10. Discussion

The present review describes a number of aspects of brain structure and function that have been studied. In brain issues characterized by low blood levels of EPA and DHA, like autism, ADHD or major depression, results intervention trials and their meta-analyses tended to be positive. Less consistent were results of trials and meta-analyses on multifactorial brain issues, like cognition. When trials determined blood levels, preferably in erythrocytes, and evaluated their results according to blood levels, changes in endpoints correlated with the changes in blood levels, and trials had clearer results when endpoints were related to blood levels reached during the trials. For trial design, these facts reinforce the results of the discussion on dietary intake vs. blood levels above, and rather clearly indicate, how future trials need to be designed, conducted, and evaluated, and also how trials should be selected for meta-analyses.

Importantly, when trial results were evaluated according to blood levels reached, they tended to fall in line with the results from the epidemiologic studies in terms of effects and effect size, e.g., results from the REDUCE-It trial were largely identical with results from Framingham [23,30]. This not only attests to widespread deficits of EPA and DHA in trial participants, but again reinforces the results of the discussion on dietary intake vs. blood levels above.

Most of the trials discussed here focus on a highly specific brain issue. However, eliminating a deficit in EPA and DHA, which is equivalent to optimizing the Omega-3 Index, reduced total mortality by some 40%, myocardial infarctions and strokes by some 50%, as demonstrated in REDUCE-It [35]. From an individual’s view, it is probably important enough to be alive without a myocardial infarction and/or stroke and/or major depression to search for and, if necessary, eliminate a deficit in EPA and DHA. A further argument might be absence of a debilitating condition like major depression or dementia. Additional improvements of highly specific brain issues would probably be considered a welcome bonus.

Some populations, like vegans and vegetarians, do not ingest sources of DHA and have been found to have a low Omega-3 Index [176]. However, also athletes have been found to have a low Omega-3 Index, probably not only due to low dietary intake, but also due to high catabolism [174,177,178,179,180]. As discussed above, pregnant women provide DHA to the fetus, which leaves most of them with a deficit in EPA and DHA [8]. Deficits in EPA and DHA in individuals with autism, autism spectrum disorder, ADHD, major depression bipolar depression and other issues of mental health have been described, as has been the benefit of reversing this deficit in most of the issues mentioned (see above). Common sense suggests determining the Omega-3 Index in the individuals and patients mentioned as a prerequisite to correct their deficit in a targeted manner.

As the individuals and patients mentioned in the previous paragraph constitute a sizable proportion of the general population, in this authors opinion, the time has come for other nations to follow the example of Statistics Canada, the Canadian national bureau of statistics. This bureau conducted a representative survey of the Canadian population, using the original method, and found an average Omega-3 Index of 4.5% [181]. In light of the relevance of health issues that come with a low Omega-3 Index for a country’s population and health system, it seems prudent for other countries to follow the Canadian example, and find out, whether a deficit in EPA and DHA exists in a country. Using extrapolations, but no direct data, the existence of a deficit has already been demonstrated for many Western and other countries [182].

There are several options, on how to increase dietary intake, and subsequently blood levels of EPA and DHA. One way that comes to mind would be increasing fish intake. Unfortunately, salmon from aquaculture, a classical source of EPA and DHA, contained half as much EPA and DHA in 2015, in comparison to 2005 [24]. In keeping, when fish distribution to employees using a work-place based catering service was increased, this increased the Omega-3 Index by only 0.55% in 4 months, not very effective [183]. Thus, current evidence points to use of food supplements. As discussed above, safety and tolerability of food supplements as sources of EPA and DHA, like oils derived from fish, krill or algae, are excellent, an Omega-3 Index < 16% provided. This indicates that the risk to balance a potential benefit of EPA and DHA against is virtually non-existent. As a consequence, the threshold to supplementing sources of Omega-3 fatty acids in order to counteract a deficit and its manifold consequences should be low.

## 11. Conclusions

Taken together, relating dietary EPA and DHA to brain functions has many uncertainties, which can be eliminated by measuring their blood levels, preferably with a standardized method in red blood cells, i.e., the Omega-3 Index providing the status of a person in terms of EPA and DHA. The perspective of the Omega-3 Index has been demonstrated to be a fruitful one for epidemiologic studies and smaller intervention trials. Evaluating results of large intervention trials from the perspective of blood levels is beginning to bear fruit as well. Thus far, the perspective of blood levels provided more convincing results than only evaluating the results by comparing EPA and DHA and placebo groups. The brain depends on a life-long supply with EPA and DHA. Evidence for of optimal blood levels of EPA and DHA in blood is strong for prevention of strokes, dementia, major depression and some brain damages, while evidence for other benefits is mounting.
